# Preparation and Evaluation of an Oral Administration System of Albendazole-Metal-Organic Framework Based on Dual Response to pH and Enzymes

**DOI:** 10.3390/ph18060819

**Published:** 2025-05-29

**Authors:** Weiqi Liu, Zhimei Guo, Yong Zhang, Yufei Guo, Ting Wang, Dahuan Liu, Chunhui Hu

**Affiliations:** 1 College of Pharmacy, Qinghai University, Xining 810001, China; ys231055001519@qhu.edu.cn (W.L.); ys221055001414@qhu.edu.cn (Z.G.); ys221055001397@qhu.edu.cn (Y.G.); 2 College of Clinical Medical, Qinghai University, Xining 810001, China; zhangyong20100507@163.com; 3 State Key Laboratory of Organic-Inorganic Composites, Beijing University of Chemical Technology, Beijing 100029, China; kongxianmei0316@163.com (T.W.); chunhuihu@hotmail.com (D.L.)

**Keywords:** albendazole, metal–organic framework, pH- and enzyme-responsive release, transmembrane transport, bioavailability

## Abstract

**Objective:** This study aims to develop a metal–organic framework (ABZ-MOFs)-based oral drug delivery system for albendazole (ABZ) to enhance its dissolution rate and oral bioavailability. **Methods:** ABZ@MOF-802, ABZ@UiO-66-NH_2_, and ABZ@MIL-125-NH_2_ were synthesized using a solvothermal method, and their physicochemical properties were characterized. The in vitro drug release was investigated under pH- and enzyme-responsive conditions, followed by transmembrane transport studies in Caco-2 cells. Finally, the oral bioavailability of ABZ@MOFs was evaluated in rats. **Results:** The particle sizes of ABZ@MOF-802, ABZ@UiO-66-NH_2_, and ABZ@MIL-125-NH_2_ were (1062.6 ± 94.8), (228.3 ± 12.3), and (502.3 ± 16.2) nm, with drug loading efficiencies of (1.71 ± 0.08%), (12.13 ± 0.04%), and (26.17 ± 0.10%), respectively. The ABZ@MOFs demonstrated structural stability in acidic environments and released ABZ under weakly acidic and neutral conditions, exhibiting distinct release profiles in the presence of different enzymes. Cellular experiments confirmed that ABZ@MOFs significantly improved transmembrane drug absorption. Pharmacokinetic analysis revealed that the bioavailability of ABZ@UiO-66-NH_2_ and ABZ@MIL-125-NH_2_ was 10.3-fold and 1.8-fold higher, respectively, compared to ABZ. **Conclusions:** The ABZ@MOFs systems effectively improved ABZ dissolution and oral bioavailability, with ABZ@UiO-66-NH_2_ showing a dual response mechanism to pH and enzymes.

## 1. Introduction

Echinococcosis is a globally prevalent zoonotic parasitic disease caused by *Echinococcus* larvae infecting humans or animals [1]. In 2015, the WHO estimated that echinococcosis led to 19,300 deaths and 871,000 disability-adjusted life years (DALYs) annually, imposing a significant public health burden [2]. The parasite primarily invades the liver, forming cysts that exert pressure on adjacent tissues and organs. Through exogenous budding, the larvae proliferate, infiltrating surrounding tissues and forming new vesicles. They can migrate via lymphatic fluid and blood to distant sites, maintaining their infectivity [3].

The pathological characteristics of echinococcosis resemble those of malignant tumors. Its prolonged incubation period, slow progression, challenging treatment, and high mortality rate have earned it the nickname “worm cancer”. Albendazole (ABZ) is the first-line treatment for echinococcosis [4,5]. ABZ disrupts the worm’s microtubule system, hindering glucose uptake and ultimately causing the larvae’s death. However, ABZ’s poor water solubility and low intestinal absorption limit its efficacy. Current oral formulations yield a clinical cure rate of less than 30% and require extended treatment durations, significantly restricting their clinical applications. Therefore, improving the solubility of albendazole and increasing the concentration of the drug within the capsule, etc., have become the main directions for the improvement of albendazole formulations. Currently, many novel formulations, including solid dispersions [6,7], synthetic salt [8], liposomes [9,10], self-microemulsions [11], and microspheres [12] have been reported to improve the absorption of ABZ, but none of these ABZ formulations have reached the clinical study stage. Among them, solid dispersions are commonly used to improve the solubility and bioavailability of drugs based on the basic melt and solvent methods, and researchers are committed to improving their preparation techniques, but the stability problem still exists [13,14]. Liposomes can encapsulate drugs with different physicochemical properties, which can improve the stability and solubility and can also improve the targeting of drugs. Liposome research started earlier; the preparation process is more mature, and it has been used in human clinics, but its clinical efficacy, production process, and other aspects are deficient [15], and it is still in the process of continuous improvement. The self-microemulsion preparation method is simple, and the stability of the original drug can be better ensured before use, which improves the utilization of the original drug of albendazole to a certain extent, but attention still needs to be paid to the safety of its main constituent components [16].

Metal–organic frameworks (MOFs) are porous materials with periodic network structures formed by coordinating inorganic metal centers with organic ligands [17]. Compared to traditional drug carriers, MOFs offer several advantages. (1) Their highly ordered crystalline structures enable the formation of uniform pores and channels, with tunable pore sizes ranging from the nanoscale to the microscale. (2) MOFs exhibit exceptionally high specific surface areas, often exceeding thousands of square meters per gram. (3) The presence of metal ions and organic ligands provides abundant active sites for interactions with drug molecules [18]. These features make MOFs highly suitable for applications requiring high drug-loading capacities.

In recent years, MOFs have emerged as a promising platform for drug delivery and are increasingly considered potential drug carriers. They offer significant improvements in drug properties. For poorly soluble small molecule drugs, MOFs with water-reactive properties can encapsulate drug molecules within nanoscale pores in an amorphous form, enhancing solubility through rapid release in dissolution media [19,20]. After drug loading, MOFs create a stable microenvironment for drugs due to strong intermolecular interactions between internal functional groups and drug molecules, protecting them from external physical and chemical influences and significantly enhancing drug stability [21].

MOFs also support sustained and controlled drug release to prevent sudden drug release and extend retention time. This control can be achieved through host/guest molecular interactions [22], surface modifications [23,24], and defect regulation [25]. Additionally, MOFs can act as stimuli-responsive carriers, reacting precisely to external triggers such as temperature, magnetic fields, ultrasound, light [26], and pH. These properties facilitate controlled drug delivery and release. Through surface modifications, MOFs can also target specific sites, enhancing drug bioavailability [27,28].

In this study, MOFs were selected as drug delivery systems for albendazole (ABZ). The chosen MOFs—MOF-802, UiO-66-NH_2_, and MIL-125-NH_2_—were evaluated for their drug-carrying capacity, in vitro release behavior, transmembrane transport efficiency in the intestinal epithelium, and ability to improve ABZ bioavailability through pharmacokinetic experiments in rats. The ultimate goal was to assess the suitability of MOFs as drug delivery systems for ABZ.

## 2. Results and Discussion

### 2.1. Particle Size, Zeta Potential, and Morphology

MOFs were synthesized via a solvothermal method, and drug encapsulation was achieved using an in situ co-crystallization technique. In this process, organic ligands, metal ions, and ABZ were combined in the reaction environment, allowing simultaneous crystallization of drugs and MOFs. Drug molecules were effectively trapped within MOF pores during crystal formation, resulting in drug-loaded ABZ@MOFs (Figure S1). The solvothermal method allows control over MOF size and morphology by adjusting the reactant ratios but is only suitable for thermally stable drugs. ABZ, with a melting point of 200 °C, meets this requirement since the reaction temperatures for synthesizing all three types of ABZ@MOFs did not exceed this threshold.

**Figure 1:**
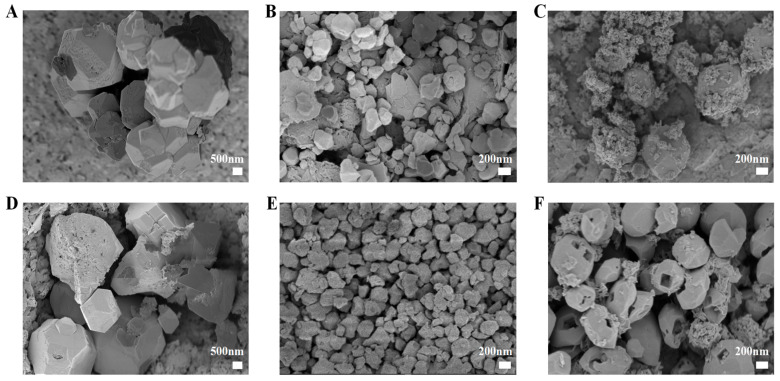
Scanning electron microscopy of MOFs and ABZ@MOFs. (**A**) MOF-802. (**B**) UiO-66-NH_2_. (**C**) MIL-125-NH_2_. (**D**) ABZ@MOF-802. (**E**) ABZ@UiO-66-NH_2_. (**F**) ABZ@MIL-125-NH_2_. Scale bars for (**A**,**D**) are 500 nm, and are 200 nm for (**B**,**C**,**E**,**F**).

**Figure 2:**
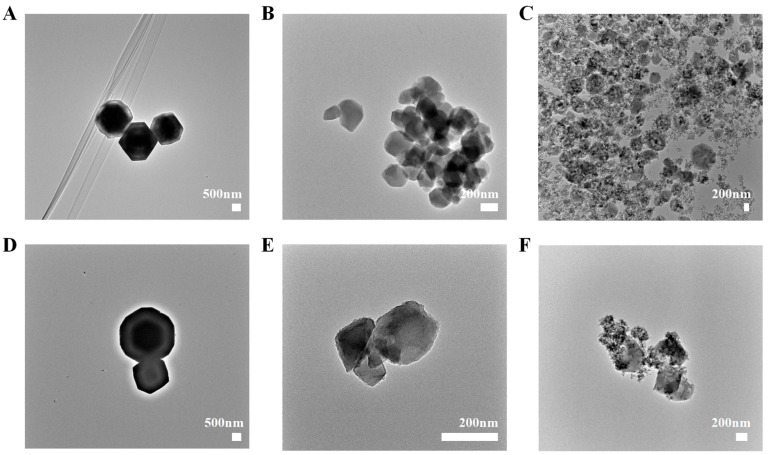
Transmission electron microscopy of MOFs and ABZ@MOFs. (**A**) MOF-802. (**B**) UiO-66-NH_2_. (**C**) MIL-125-NH_2_. (**D**) ABZ@MOF-802. (**E**) ABZ@UiO-66-NH_2_. (**F**) ABZ@MIL-125-NH_2_. Scale bars for (**A**,**D**) are 500 nm, and are 200 nm for (**B**,**C**,**E**,**F**).

SEM images (Figure 1) and TEM images (Figure 2) revealed distinct morphological characteristics for the synthesized ABZ@MOFs. ABZ@MOF-802 exhibited a spheroidal polyhedral shape with well-defined edges and a smooth surface, maintaining its morphology after drug loading. UiO-66-NH_2_ displayed an irregular polyhedral shape with a smooth surface and uniform particle size distribution following drug loading. MIL-125-NH_2_ formed rhombohedral dodecahedrons with angular edges. Impurity particles adhered to the surface both before and after drug loading, and distinct square pore structures were visible on the surface of ABZ@MIL-125-NH_2_.

**Figure 3:**
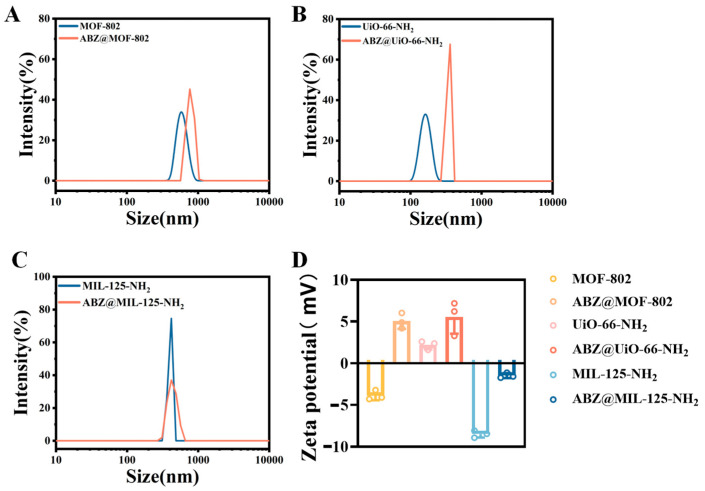
Particle size and Zeta potential of MOF-802 and ABZ@MOFs. Particle size distribution for (**A**) MOF-802 and ABz@MOF-802, (**B**) UiO-66-NH_2_ and ABZ@UiO-66-NH_2_, (**C**) MIL-125-NH_2_ and ABZ@MIL-125-NH_2_. (**D**) Zeta Potential of MOFs and ABZ@MOFs.

The particle sizes of synthesized MOF-802, UiO-66-NH_2_, and MIL-125-NH_2_ were (793.4 ± 22.4), (159.8 ± 11.3), and (425.9 ± 5.0) nm, respectively. Following drug loading, the sizes increased to (1062.6 ± 94.8), (228.3 ± 12.3), and (502.3 ± 16.2) nm. The Zeta potentials of MOF-802, UiO-66-NH_2_, and MIL-125-NH_2_ were (−3.9 ± 0.6), (2.2 ± 0.5), and (−6.8 ± 0.7) mV, respectively. After drug loading, the potentials increased to (5.0 ± 0.9), (5.6 ± 0.2), and (−3.3 ± 0.4) mV. ABZ is a weakly alkaline drug that dissociates into positively charged ions in weakly acidic environments, such as acetic or formic acid solutions used during synthesis. This dissociation increased the Zeta potential of ABZ-loaded MOFs (Figure 3D).

### 2.2. Characterization of ABZ@MOFs

**Figure 4:**
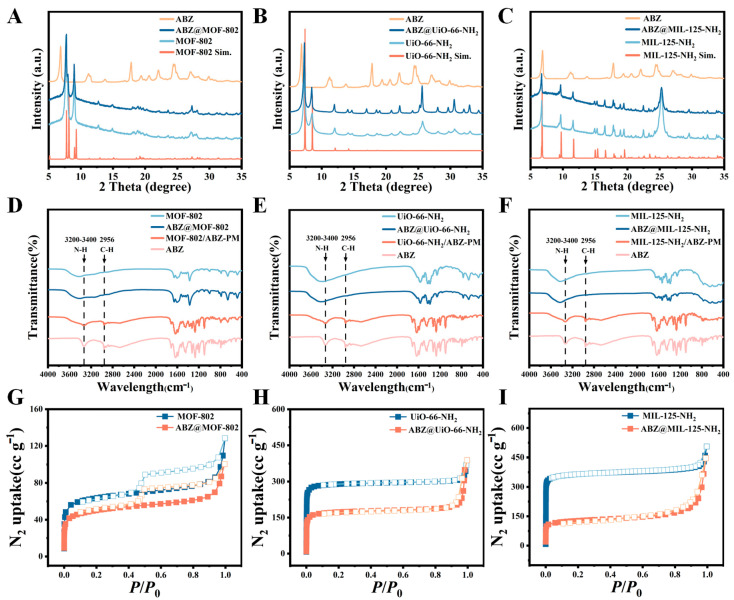
Comprehensive Characterization of MOFs and ABZ@MOFs. (**A**–**C**) Powder X-ray diffraction patterns. (**D**–**F**) FT-IR spectra of ABZ, MOFs, ABZ@MOFs, and MOFs/ABZ-PM. (**G**–**I**) N_2_ adsorption/desorption isotherms.

The crystalline structure of the synthesized materials was examined using powder X-ray diffraction (PXRD) (Figure 4A–C). The diffraction peaks for MOF-802 were observed at 2θ 7.77°, 8.98°, 12.75°, and 14.95°, matching the PXRD pattern of simulated MOF-802. UiO-66-NH_2_ exhibited high-intensity, sharp diffraction peaks, with characteristic reflections at 2θ 7.32°, 8.46°, 12.08°, and 14.18°, indicating good crystallinity and structural stability. MIL-125-NH_2_ demonstrated prominent peaks at 2θ 6.71°, 9.69°, and 11.61°, which were consistent with the simulated spectra. These results confirmed the successful synthesis of the three MOFs. Notably, the diffraction peak positions remained unchanged after ABZ loading, indicating that the crystal structures were preserved.

The FT-IR spectra provided further insights into the chemical composition of ABZ, MOFs, ABZ@MOFs, and the physical mixture MOFs/ABZ-PM (Figure 4D–F). In MOF-802, a characteristic Zr-O stretching vibration appeared at 663 cm^−1^, while the peaks at 1371 cm^−1^ and 1657 cm^−1^ corresponded to the stretching vibrations of C-O and C=O, respectively. The broad absorption around 3400 cm^−1^ was attributed to -OH stretching. For UiO-66-NH_2_, Zr-O bond vibrations were observed at 665 cm^−1^ and 767 cm^−1^, while aromatic C=C bond stretching in 2-amino-terephthalic acid appeared at 1600–1500 cm^−1^. A broad peak between 3700 and 3000 cm^−1^ was associated with carboxylic acid -OH stretching. MIL-125-NH_2_ exhibited characteristic Ti-O and O-Ti-O vibrations between 600 and 1000 cm^−1^, with a strong -OH absorption at 3400 cm^−1^.

ABZ displayed distinct C-H and N-H stretching peaks at 2956 cm^−1^ and 3313 cm^−1^, respectively. However, these characteristic ABZ peaks disappeared in the ABZ@MOFs spectra, indicating encapsulation within the MOF frameworks via chemical coordination bonds rather than simple physical mixing. The consistent spectra before and after drug loading suggested minimal structural changes to the MOFs. The reduced response to FT-IR may have resulted from the shielding effect of the MOF pores or weak ABZ absorption after encapsulation [29].

The porosity of the MOFs was examined using N_2_ adsorption/desorption isotherms (Figure 4G–I). MOF-802 and ABZ@MOF-802 exhibited type IV adsorption isotherms with hysteresis loops, indicating the coexistence of micropores and mesopores. Both UiO-66-NH_2_ and MIL-125-NH_2_ displayed typical type I isotherms, characteristic of microporous materials. Drug loading led to a reduction in the BET specific surface area (Table 1), confirming successful ABZ encapsulation within the MOFs.

**Figure 5:**
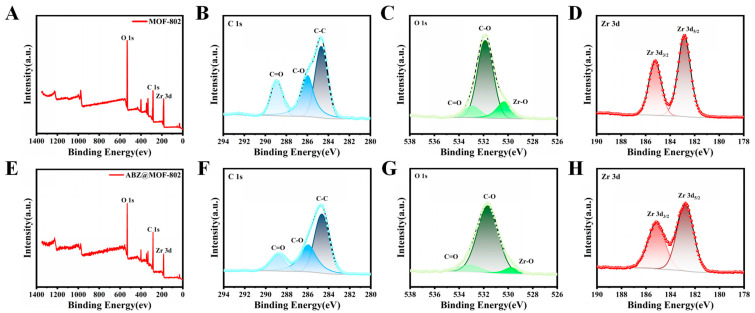
XPS analysis of MOF-802 and ABZ@MOF-802. (**A**,**E**) General XPS spectra. High-resolution XPS spectra for (**B**,**F**) C 1s, (**C**,**G**) O 1s, and (**D**,**H**) Zr 3d before and after ABZ loading.

Figure 5 presents the full XPS spectra and high-resolution elemental analyses of MOF-802 before and after ABZ loading. The characteristic peaks at 532 eV, 285 eV, and 184 eV correspond to the presence of O, C, and Zr elements, respectively. In the C 1s spectra (Figure 5B,F), the peaks at 284.8 eV, 286 eV, and 288.8 eV are attributed to C-C and carboxyl carbon (C-O and C=O) bonds. Analysis of the Zr 3d orbital spectra (Figure 5D,H) reveals two distinct peaks at 182.8 eV and 185.2 eV, corresponding to Zr 3d_5/2_ and Zr 3d_3/2_, with a peak spacing of 2.4 eV. This confirms that Zr remains in the +4 oxidation state, even after ABZ loading.

**Figure 6:**
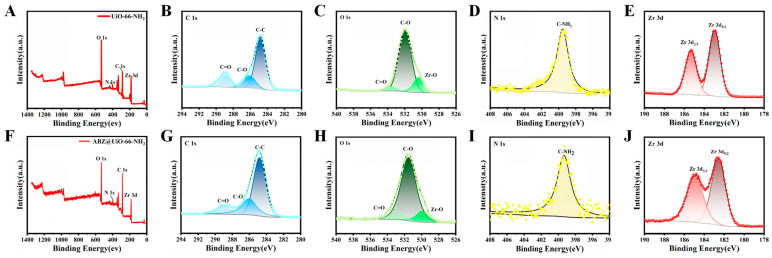
XPS analysis of UiO-66-NH_2_ and ABZ@UiO-66-NH_2_. (**A**,**F**) General XPS spectra. High-resolution XPS spectra for (**B**,**G**) C 1s, (**C**,**H**) O 1s, (**D**,**I**) N 1s, and (**E**,**J**) Zr 3d before and after ABZ loading.

The XPS results for UiO-66-NH_2_ and ABZ@UiO-66-NH_2_ are depicted in Figure 6. The full spectra (Figure 6A,F) confirm the presence of O, C, N, and Zr, with characteristic peaks at 532 eV, 400 eV, 285 eV, and 184 eV, respectively. High-resolution C 1s spectra (Figure 6B,G) calibrated at 284.8 eV exhibit peaks corresponding to C-C, C-O, and C=O bonds. The O 1s spectra (Figure 6C,H) display absorption peaks for Zr-O, C-O, and C=O at 530.4 eV, 532 eV, and 533.6 eV, respectively. After ABZ loading, these peaks shift toward lower binding energies by 0.5 eV and 0.7 eV for the C-O and C=O bonds, respectively, indicating the involvement of carboxyl groups in drug binding. The Zr 3d spectra (Figure 6E,J) also exhibit shifts toward lower binding energy, suggesting chemical interactions between ABZ and the MOF framework.

**Figure 7:**
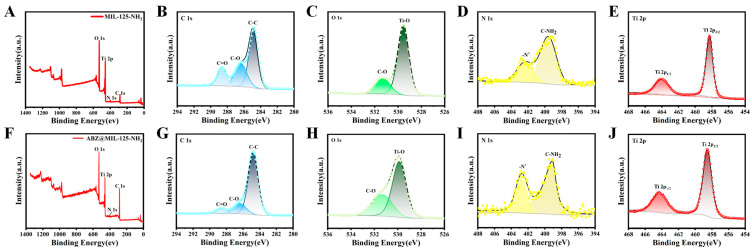
XPS analysis of MIL-125-NH_2_ and ABZ@MIL-125-NH_2_. (**A**,**F**) General XPS spectra. High-resolution XPS spectra for (**B**,**G**) C 1s, (**C**,**H**) O 1s, (**D**,**I**) N 1s, and (**E**,**J**) Ti 2p before and after ABZ loading.

As illustrated in Figure 7A,F, MIL-125-NH_2_ comprises C, O, N, and Ti elements. The XPS analysis reveals that the Ti 2p spectra exhibit a characteristic bimodal pattern, with peaks at 464 eV and 458.3 eV corresponding to Ti 2p_1/2_ and Ti 2p_3/2_, respectively. These peaks are indicative of Ti^4+^. After ABZ loading, the binding energies shift to 464.3 eV and 458.5 eV, suggesting chemical interactions between ABZ and the framework. The N 1s spectra (Figure 7D) of MIL-125-NH_2_ display peaks at 399.6 eV and 402.4 eV, corresponding to -NH_2_ and -N^+^ groups, respectively [30]. After ABZ encapsulation, these peaks shift to 399.1 eV and 402.8 eV, implying possible interactions between ABZ and amino groups within the framework.

Interestingly, no S element characteristic of ABZ is detected in the XPS spectra of ABZ@MOFs. This absence is likely due to the limited detection depth of XPS (approximately 10 nm) and the encapsulation method used for ABZ loading. These findings strongly suggest that ABZ is embedded within the MOF skeleton rather than merely adsorbed on its surface.

**Figure 8:**
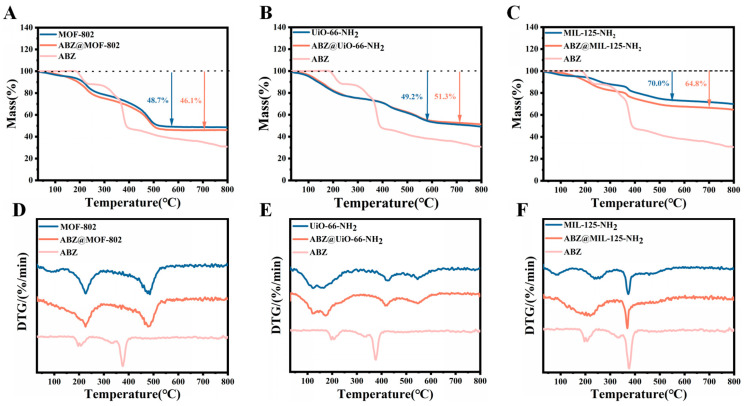
Thermal stability assessment. Evaluation through thermogravimetric (TGA) and differential thermal gravimetric (DTG) analyses. (**A**,**D**) TGA and DTG curves of MOF-802 and ABZ@MOF-802; (**B**,**E**) UiO-66-NH_2_ and ABZ@UiO-66-NH_2_; (**C**,**F**) MIL-125-NH_2_ and ABZ@MIL-125-NH_2_.

Thermal stability analysis (Figure 8) demonstrates distinct thermal behaviors for MOFs and their ABZ-loaded counterparts. For MOF-802 and ABZ@MOF-802 (Figure 8A,D), both exhibit stability up to 150 °C. Between 200 °C and 500 °C, a significant mass loss of approximately 45% is observed, followed by stabilization between 500 °C and 800 °C. The decomposition in this range is attributed to the breakdown of organic ligands.

UiO-66-NH_2_ and ABZ@UiO-66-NH_2_ (Figure 8B,E) exhibit pronounced weight loss between 30 °C and 200 °C, likely due to the desorption of surface water molecules and the evaporation of residual acetic acid solvent. A faster mass loss occurs between 380 °C and 450 °C, associated with the decomposition of organic ligands. Between 500 °C and 600 °C, an additional 8% weight loss is attributed to the collapse of the framework and transformation of Zr-O clusters into ZrO_2_ metal oxide. The mass stabilizes beyond 600 °C [31].

TGA analysis of MIL-125-NH_2_ and ABZ@MIL-125-NH_2_ (Figure 8C,F) reveals three distinct phases. The first stage, from 30 °C to 300 °C, corresponds to the desorption of surface-adsorbed water and the evaporation of residual methanol and DMF solvents. The second stage, between 300 °C and 400 °C, is marked by the decomposition of ABZ and the collapse of the framework due to the breakdown of metal coordination bonds. Beyond 400 °C, the structure stabilizes as it transforms into TiO_2_ [32], with final residual masses of 70.0% for MIL-125-NH_2_ and 64.8% for ABZ@MIL-125-NH_2_.

### 2.3. Drug Loading and Responsive Release of ABZ@MOFs

The drug loading capacity of ABZ@MOFs was assessed by UV-spectrophotometry. The drug loading content for ABZ@MOF-802, ABZ@UiO-66-NH_2_, and ABZ@MIL-125-NH_2_ were determined to be (1.71 ± 0.08)%, (12.13 ± 0.04)%, and (26.17 ± 0.10)%, respectively. The encapsulation efficiency for ABZ@MOF-802, ABZ@UiO-66-NH_2_, and ABZ@MIL-125-NH_2_ were determined to be (40.5 ± 0.15)%, (91.4 ± 0.70)%, and (56.0 ± 0.11)%, respectively. The N₂ adsorption/desorption measurements revealed that both UiO-66-NH_2_ and MIL-125-NH_2_ exhibited higher BET-specific surface areas and larger total pore volumes compared to MOF-802, which likely facilitates greater drug accommodation. Additionally, the -NH₂ groups on the structures of UiO-66-NH_2_ and MIL-125-NH_2_ may facilitate drug loading through hydrogen bond interactions with albendazole.

**Figure 9:**
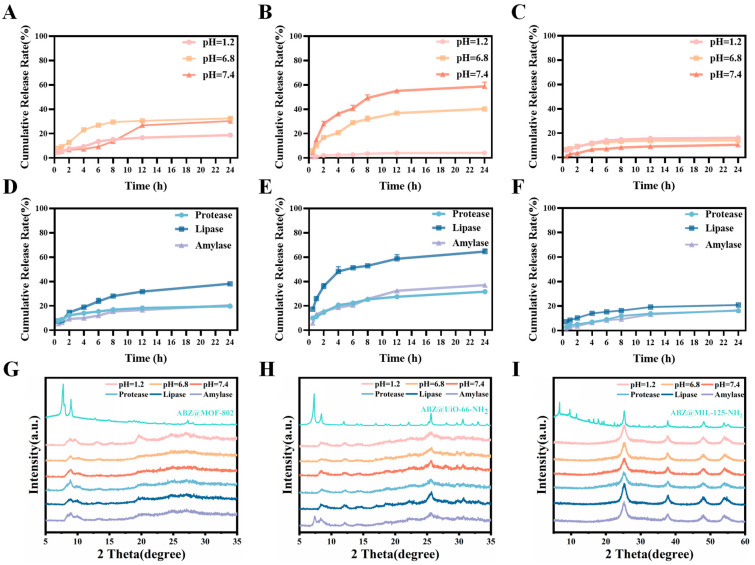
Simulated in vitro drug release profiles of ABZ@MOFs under various conditions. (**A**–**C**) Drug release from ABZ@MOF-802, ABZ@UiO-66-NH_2_, and ABZ@MIL-125-NH_2_ at pH 1.2, 6.8, and 7.4. (**D**–**F**) Drug release profiles in the presence of protease, lipase, and amylase for ABZ@MOF-802, ABZ@UiO-66-NH_2,_ and ABZ@MIL-125-NH_2_. (**G**–**I**) PXRD patterns of ABZ@MOF-802, ABZ@UiO-66-NH_2_, and ABZ@MIL-125-NH_2_ after 24 h of release in different media.

Oral administration remains the most common and simplest drug delivery route. However, various physiological barriers, such as the gastrointestinal tract, significantly hinder oral drug absorption, resulting in low bioavailability. To simulate the physiological conditions of the stomach, intestine, and normal body fluids, hydrochloric acid solution (pH 1.2) and phosphate-buffered saline (PBS) solutions at pH 6.8 and 7.4 were selected as release media. The drug release profiles are shown in Figure 9A–C. ABZ@MOF-802 demonstrated stable drug release under weakly acidic and neutral conditions, while it remained stable under strongly acidic conditions. ABZ@MIL-125-NH_2_ exhibited a cumulative release rate of less than 20% after 24 h under all three pH conditions, indicating a steady and controlled release profile.

In contrast, ABZ@UiO-66-NH_2_ exhibited minimal release under acidic conditions (less than 5% after 24 h), suggesting stability in the gastric environment. In PBS at pH 6.8, a faster release occurred, with the cumulative release reaching over 40%, while at pH 7.4, the release rate increased further, achieving a cumulative release of (62.4 ± 0.70)% within 24 h. These results highlight the pH-responsive release behavior of the three ABZ@MOFs. Additionally, a preliminary experiment indicated that ABZ@MOFs released more drugs in intestinal homogenate, likely due to the influence of digestive enzymes on the release behavior. To further investigate this, enzymes abundant in the intestine—trypsin (protease), pancreatic lipase, and α-amylase—were employed. As shown in Figure 9D–F, ABZ@UiO-66-NH_2_ exhibited superior drug release under both varying pH conditions and enzyme presence. Among the three enzymes, lipase had the most significant effect on the drug release from ABZ@MOFs.

The in vitro drug release data were analyzed using first-order, zero-order, and Higuchi models to understand the release kinetics of ABZ@MOFs. The results, detailed in Tables S1–S3, indicate that the first-order release model provided the best fit, with R^2^ values closest to 1 for each formulation under different pH conditions, suggesting that drug release primarily follows a diffusion-driven process. Initially, there is a rapid release rate, followed by a gradual decrease over time, leading to a more stable and sustained release. Under the influence of enzymes, the Higuchi model provided the best fit, indicating that drug release, in this case, is governed by a combination of diffusion and framework dissolution. In this scenario, drug molecules diffuse through the pores or channels of the MOF, with an initial rapid release followed by an accelerated release phase while maintaining a relatively steady release trend throughout. This release profile enables the drug to reach therapeutic concentrations quickly and maintain an effective level over time, improving bioavailability and therapeutic efficacy. These findings suggest that all three ABZ@MOFs maintained the stability of the drug delivery system in the stomach, facilitating ABZ’s passage into the intestine, where the drug was released in response to enzymatic activity, thereby exerting its therapeutic effect. However, ABZ@UiO-66-NH_2_ exhibited the most efficient release.

To further elucidate the release behaviors, the PXRD patterns of ABZ@MOFs after release under different conditions were analyzed (Figure 9G–I). The PXRD patterns of ABZ@MOF-802 and ABZ@UiO-66-NH_2_ completely collapsed after release, which corresponds to a higher drug release. In contrast, the crystal structure of ABZ@MIL-125-NH_2_ remained intact, even after 24 h of release under different conditions, which could explain its lower drug release capability. Furthermore, phosphate ions (PO_4_^3−^), abundant in the physiological environment, likely play a role in disrupting the MOF structure by exchanging phosphate groups with carboxylate ligands, leading to framework degradation and drug release. The stronger release observed for the two Zr-based MOFs compared to MIL-125-NH_2_ may be due to the higher binding affinity between Zr and phosphate ions compared to Ti and phosphate ions.

Due to the low drug loading (1.71%) and large particle size of ABZ@MOF-802, it was excluded from subsequent cell and animal experiments.

### 2.4. Study of Transmembrane Transport In Vitro

ABZ@UiO-66-NH_2_ and ABZ@MIL-125-NH_2_ on Caco-2 cells were evaluated using the CCK-8 method (Figure S1). At drug concentrations above 20 μg·mL^−1^, cell survival rates decreased to below 80%. The following concentrations were tested: 20 μg·mL^−1^ (high), 10 μg·mL^−1^ (medium), and 5 μg·mL^−1^ (low).

**Figure 10:**
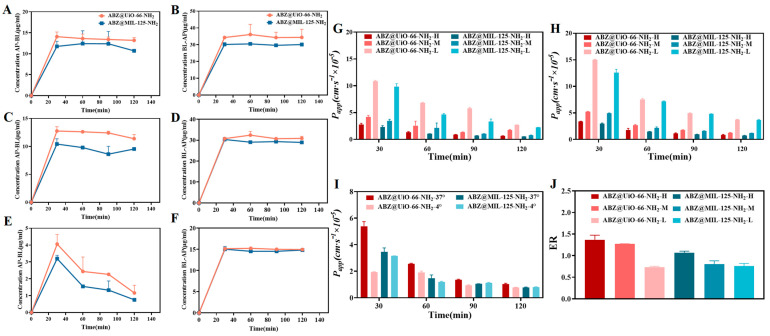
Transmembrane transport studies in Caco-2 cell models. (**A**,**B**) Transmembrane concentration on the AP-BL side for high concentration ABZ@MOFs. (**C**,**D**) Transmembrane concentration on the AP-BL side for medium concentration ABZ@MOFs. (**E**,**F**) Transmembrane concentration on the AP-BL side for low concentration ABZ@MOFs. (**G**) Transmembrane transport efficiency (*P_app_*) on the AP-BL side at high, medium, and low concentrations of ABZ@MOFs. (**H**) Transmembrane transport efficiency (*P_app_*) on the BL-AP side at high, medium, and low concentrations of ABZ@MOFs. (**I**) Transmembrane transport efficiency (*P_app_*) at 37 °C and 4 °C. (**J**) Efflux ratio (ER) at high, medium, and low concentrations of ABZ@MOFs.

The transmembrane transport volume and permeability (*P_app_*) of ABZ@UiO-66-NH_2_ and ABZ@MIL-125-NH_2_ from the apical (AP) side to the basolateral (BL) side, as well as from the BL side to the AP side, in a Caco-2 monolayer cell model at varying concentrations, are shown in Figure 10. As illustrated in Figure 10A–H, the drug concentration and permeability of ABZ@UiO-66-NH_2_ were higher than those of ABZ@MIL-125-NH_2_ at all concentrations (high, medium, and low) on both the AP and BL sides. This suggests that ABZ@UiO-66-NH_2_ possesses superior transmembrane transport capacity compared to ABZ@MIL-125-NH_2_. Intestinal epithelial cells represent a key barrier to the absorption of orally administered drug carriers. The positively charged ABZ@UiO-66-NH_2_ is more readily absorbed by the negatively charged membranes of these cells. Additionally, the physical properties of drug carriers, including size, shape, and rigidity, significantly influence their absorption in the small intestine [33,34]. Nano-formulations typically have a particle size range of 10–200 nm [35], which enhances the physicochemical properties of drugs, improving their solubility and permeability [36]. The particle size of ABZ@UiO-66-NH_2_ is 228.3 nm, smaller than that of ABZ@MIL-125-NH_2_, allowing it to more easily cross the intestinal epithelium and reach its target site for therapeutic action.

To further investigate the transmembrane transport mechanism of ABZ@UiO-66-NH_2_ and ABZ@MIL-125-NH_2_, their transport across the AP-BL side in the Caco-2 monolayer cell model was examined at two different temperatures (4 °C and 37 °C). The results are presented in Figure 10I. The *P_app_* of ABZ@UiO-66-NH_2_ decreased over time at both temperatures, with lower *P_app_* values observed at 4 °C compared to 37 °C. These findings indicate that transmembrane transport is temperature-dependent, suggesting an energy-dependent process. The drug transport mechanism can also be inferred from the efflux ratio (ER). When the ER is less than 1, passive transport predominates. An ER between 1 and 1.5 indicates a combination of passive and active transport. An ER greater than 1.5 suggests the involvement of efflux proteins. As shown in Figure 10J, the ER of ABZ@UiO-66-NH_2_ at high and medium concentrations ranged from 1 to 1.5, while the ER of ABZ@MIL-125-NH_2_ at medium and low concentrations was less than 1. These results suggest that ABZ@UiO-66-NH_2_ follows a transmembrane transport mode involving passive diffusion and active transport, while ABZ@MIL-125-NH_2_ primarily relies on passive transport.

### 2.5. In Vivo Pharmacokinetic Study

**Figure 11:**
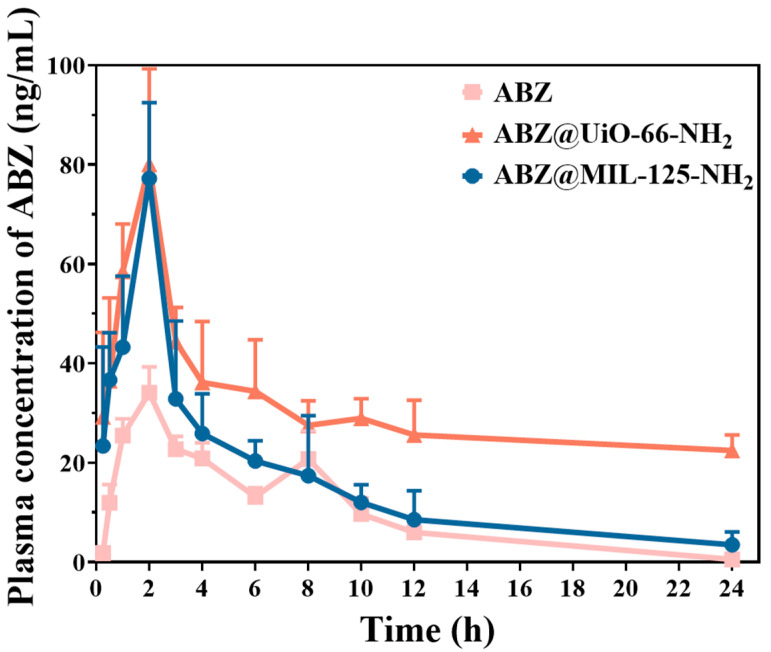
In vivo pharmacokinetics of ABZ, ABZ@UiO-66-NH_2_, and ABZ@MIL-125-NH_2_.

Following intragastric administration of ABZ and ABZ@MOFs in rats (Figure 11 and Table 2), the results revealed a significant increase in the area under the blood concentration-time curve (AUC_0–∞_) for ABZ@UiO-66-NH_2_ and ABZ@MIL-125-NH_2_. Specifically, the AUC_0–∞_ of ABZ@UiO-66-NH_2_ was 2269.66 ± 707.32 μg·L^−1^·h, which is 10.3 times higher than that of ABZ API (219.91 ± 25.27 μg·L^−1^·h). As shown in the figure, the blood concentration of ABZ@UiO-66-NH_2_ remained higher than that of ABZ within 24 h post-administration. The half-life of ABZ@UiO-66-NH_2_ was prolonged, and the Mean Residence Time (MRT_0–∞_) in vivo was extended, resulting in a sustained increase in plasma concentration and improved bioavailability of ABZ. In contrast, the AUC_0–∞_ of ABZ@MIL-125-NH_2_ was 1.8 times greater than that of ABZ. Although the peak blood concentration (*C*_max_) of ABZ@MIL-125-NH_2_ was higher than that of ABZ, its clearance rate was significantly enhanced, leading to faster metabolism and more rapid excretion from the bloodstream. This suggests that ABZ may not have been immediately released from the formulation. These findings indicate that the MOF formulations of ABZ effectively enhance its oral bioavailability.

## 3. Materials and Methods

### 3.1. Materials

Zirconium oxychloride octahydrate (ZrOCl_2_·8H_2_O, LOT: DMX553, 99%), 2-aminobenzene-1,4-dicarboxylic acid (CAS: 10312-55-7, 99%), and 3,5-pyrazoledicarboxylic acid (C_5_H_6_N_2_O_5_, LOT: DNZ257, 97%) were obtained from Bidepharm, Shanghai, China. *N*,*N*-Dimethylformamide (DMF, >99%) and acetic acid (CH_3_COOH, >99%) were purchased from Tianjin Fuyu Fine Chemical Co., Ltd., Tianjin, China. Titanium (IV) isopropoxide (Ti_4_(OCH_3_)_16_, LOT: LA60V1, 98%) was supplied by J&K Scientific Co., Ltd., Beijing, China. Formic acid (HCOOH, CAS: 64-18-6, 98%, AR), albendazole (CAS: 54965-21-8, >98%), and hydrochloric acid (HCl, LOT: 7647-01-0) were sourced from MREDA Technology Co., Ltd., Beijing, China. Methanol (CH_3_OH, LOT: 67-56-1) and anhydrous ethanol (CH_3_CH_2_OH, LOT: 64-17-5) were provided by Tianjin Damao Chemical Reagent Co., Ltd., Tianjin, China. PBS (pH 7.4, LOT: WHB824A241) was purchased from Wuhan Pricella Biotechnology Co., Ltd., Wuhan, China. Pancreatic Protease (JS249293), Pancreatic Lipase (JS256398), and α-Amylase (F14IS206584) were all purchased from Shanghai Yuan Ye Biotechnology Co., Ltd., Shanghai, China. Sodium Carboxymethylcellulose (CMC-Na, CAS: 9004-32-4) purchased from Shanghai McLin Biochemical Technology Co., Ltd., Shanghai, China.

### 3.2. Instrument

Zetasizer Nano ZS 90 model, Malvern laser particle size analyzer (Malvern Instruments Ltd., Worcestershire, England, UK), JSM-7900F, field emission scanning electron microscope (JEOL Ltd., Tokyo, Japan), H-600, field emission transmission electron microscope (Hitachi Ltd., Tokyo, Japan), ESCALAB™Xi+, powder X-ray diffractometer (Thermo Fisher Scientific Inc., Waltham, MA, USA), Nicolet 6700, infrared spectrometer (Thermo Electron Corporation, Waltham, MA, USA), ESCALAB Xi+, multifunctional X-ray photoelectron spectrometer (Thermo Scientific Inc., Waltham, MA, USA), BSD-PS2 surface area and pore size analyzer (Beijing BESIDe Instrument Technology Co., Ltd., Beijing, China), STA449 F3, simultaneous thermal analysis instrument (Netzsch GmbH, Munich, Germany), UVmini-1280, ultraviolet-visible spectrophotometer (Shimadzu Corporation, Kyoto, Japan), e2695 high-performance liquid chromatograph (Waters Corporation, Milford, MA, USA), UHPLC-MS/MS (UltiMate 3000 and Q-Exactive Orbitrap MS, Thermo Scientific Inc., Waltham, MA, USA).

### 3.3. Cell Lines and Experimental Animals

Caco-2 cells (Catalog No.: CL-0050) were purchased from Wuhan Promega Life Science Technology Co., Ltd. (Wuhan, China). SPF grade Wistar rats were obtained from SiPeiFu (Beijing) Biotechnology Co., Ltd. (Beijing, China). The rats were 4–6 weeks old with a body weight of 180–200 g; half were male and the other half were female.

### 3.4. Synthesis of MOF-802 and ABZ@MOF-802

MOF-802: ZrOCl_2_·8H_2_O (8.7 mmol, 2.8 g) and 3,5-pyrazoledicarboxylic acid (10 mmol, 1.74 g) was dissolved in 30 mL of N,N-DMF and 20 mL of formic acid. The mixture was stirred at room temperature for 20 min. Subsequently, the solution was transferred to a reactor and heated at 130 °C for 3 days. After cooling to room temperature, the mixture was filtered, and the solid product was collected, washed three times with deionized water, and dried under vacuum at 80 °C for 24 h.

ABZ@MOF-802: ZrOCl_2_·8H_2_O (8.7 mmol, 2.8 g) and 3,5-pyrazoledicarboxylic acid (10 mmol, 1.74 g) was added to 30 mL of N,N-DMF. Meanwhile, 200 mg of albendazole was dissolved in 20 mL of formic acid. Both mixtures were stirred for 20 min at room temperature, then combined and transferred to a reactor. The reaction was carried out at 130 °C for 3 days. After cooling to room temperature, the mixture was filtered, and the solid product was collected. It was washed three times with deionized water and dried under vacuum at 80 °C for 24 h.

### 3.5. Synthesis of UiO-66-NH_2_ and ABZ@UiO-66-NH_2_

UiO-66-NH_2_: ZrOCl_2_·8H_2_O (850 mg, 2.6 mmol) and 2-aminobenzene-1,4-dicarboxylic acid (457 mg, 2.5 mmol) were added to a round-bottom flask containing 10 mL of deionized water. To this, 20 mL of acetic acid was slowly added while stirring on a magnetic stirrer for 10 min. The mixture was then refluxed in an oil bath at 105 °C for 24 h. After the reaction, the solid product was collected by filtration, washed three times each with deionized water and ethanol, and dried at 100 °C.

ABZ@UiO-66-NH_2_: ZrOCl_2_·8H_2_O (850 mg, 2.6 mmol) and 2-aminobenzene-1,4-dicarboxylic acid (457 mg, 2.5 mmol) were added to a round-bottom flask with 10 mL of deionized water. Separately, 200 mg of albendazole was dissolved in 20 mL of acetic acid, and the resulting solution was added to the flask. The mixture was stirred on a magnetic stirrer for 10 min and then refluxed in an oil bath at 105 °C for 24 h. The resulting solid was collected by filtration, washed three times with deionized water and ethanol, and dried at 100 °C.

### 3.6. Synthesis of MIL-125-NH_2_ and ABZ@MIL-125-NH_2_

MIL-125-NH_2_: A total of 2-aminobenzene-1,4-dicarboxylic acid (6 mmol, 1.086 g) was dissolved in a mixture of 25 mL methanol (CH_3_OH) and 25 mL N,N-DMF. To this solution, 10 mL of acetic acid was added with stirring to ensure complete dissolution. Subsequently, Titanium (Ⅳ) isopropoxide (3 mmol) was added under continuous stirring. The resulting mixture was transferred to a reactor and heated at 433 K for 48 h. After cooling, the yellowish product was filtered, washed with DMF to remove unreacted materials, and subjected to solvent exchange using methanol. Finally, the sample was activated under vacuum at 393 K overnight, and the solid product was collected.

ABZ@MIL-125-NH_2_: To prepare ABZ-loaded MIL-125-NH_2_, 2-aminobenzene-1,4-dicarboxylic acid (6 mmol, 1.086 g) was dissolved in a mixture of 25 mL methanol and 25 mL DMF. Separately, 200 mg of albendazole was dissolved in 10 mL acetic acid, and this solution was added to the mixture. Titanium (Ⅳ) isopropoxide (3 mmol) was then introduced under stirring. The solution was transferred to a reactor and heated at 433 K for 48 h. After cooling, the yellowish product was filtered and washed with DMF to eliminate unreacted materials, followed by solvent exchange with methanol. The sample was then activated under vacuum at 393 K overnight, and the final solid product was collected.

### 3.7. Particle Size and Zeta Potential

The synthesized MOFs and ABZ@MOFs were collected and dispersed in methanol using ultrasonic agitation to ensure uniform suspension. The particle sizes and zeta potentials of each sample were measured using a Malvern laser particle size analyzer (Zetasizer Nano Model ZS 90). Each measurement was performed in triplicate to ensure accuracy and reproducibility.

### 3.8. Morphological Characterization

The dried ABZ@MOFs and blank MOFs were dispersed in anhydrous ethanol using ultrasonic dispersion. The resulting dispersions were then dropwise applied to a carbon copper mesh film, dried, and examined under a field scanning transmission electron microscope (JSM-7900F) and field emission transmission electron microscope (H-600).

### 3.9. Powder X-Ray Diffraction (PXRD)

The crystalline structure of the samples was characterized using a powder X-ray diffractometer (ESCALAB™Xi+) with Cu-Kα radiation. The scanning range was set to 2θ = 5–35° and 5–60°, with a scanning speed of 10°/min and a step size of 0.02°/2θ.

### 3.10. Fourier Transform Infrared Spectroscopy (FT-IR)

FT-IR spectroscopy was used to analyze the major functional groups in the MOF formulations. Dried ABZ, MOFs, and ABZ@MOFs were mixed with KBr in a specific ratio, milled, and pressed into a pellet. The sample was then analyzed using an infrared spectrometer (Nicolet 6700) with the following instrumental parameters: spectral resolution of 4 cm^−1^ and a scanning range of 500–4000 cm^−1^.

### 3.11. N_2_ Adsorption-Desorption Isotherms

The samples were dried in a vacuum oven at 80 °C for 24 h. The N_2_ adsorption/desorption isotherms of the MOFs and ABZ@MOFs were measured at 77 K using a BSD-PS2 specific surface area and pore size analyzer. The specific surface areas and pore size distributions were determined and calculated based on the obtained isotherms.

### 3.12. X-Ray Photoelectron Spectroscopy (XPS)

XPS was employed to detect the surface elemental compositions of solid dispersion particles using the ESCALAB 250Xi equipped with 200 W monochromatic Al Kα radiation. Full spectrum scanning: step 1 eV, pass 100 eV, dwell time 50 ms. Element scanning: step 0.1 eV, pass energy 50 eV, dwell time 100 ms, scan times 10 times. Spectral analysis was conducted using Avantage software (v5.9921), and curve fitting was performed using Origin 2022 software. The relative atomic concentrations in blends were calculated based on the integral peak intensities and sensitivity factors provided by the manufacturer. The fraction of each component at the surface was calculated from the chemical formulas and the characteristic elements of the drug and polymer.

### 3.13. Thermal Stability Analysis

The thermal stability of the preparation was evaluated by a synchronous thermal analyzer. The temperature rise rate from 30 °C to 800 °C at 10 °C⸱cm^−1^ under a nitrogen atmosphere was recorded to record the relationship between mass loss percentage and temperature.

### 3.14. Drug Loading Content (DL) and Encapsulation Efficiency (EE)

To determine the drug loading capacity, 5 mg of ABZ@MOFs was precisely weighed and dissolved in 5 mL of a hydrochloric acid and PBS mixture (*v*/*v* = 1:1). The solution was allowed to stand for 48 h to ensure complete dissolution. The absorbance of ABZ was then measured at a wavelength of 295 nm using a UV-visible spectrophotometer (UVmini-1280). The drug loading content was calculated using the following formula:

(1) $$\mathrm{DL}\;\left(\%\right)=\frac{\mathrm{Amount}\;\mathrm{of}\;\mathrm{encapsulated}\;\mathrm{ABZ}}{\mathrm{Total}\;\mathrm{amount}\;\mathrm{of}\;\mathrm{ABZ}@\mathrm{MOFs}}\times 100\%$$

(2) $$\mathrm{EE}\;\left(\%\right)=\frac{\mathrm{Amount}\;\mathrm{of}\;\mathrm{encapsulated}\;\mathrm{ABZ}}{\mathrm{Total}\;\mathrm{amount}\;\mathrm{of}\;\mathrm{ABZ}}\times 100\%$$

### 3.15. In Vitro Release Rate

The release of ABZ@MOFs at different pH was investigated. Hydrochloric acid solution with pH 1.2 and PBS solution with pH 6.8 and 7.4 were used to simulate the physiological environment of the stomach, intestine, and normal body fluids, respectively. A total of 3 mg ABZ@MOFs was dissolved in 35 mL hydrochloric acid solution or PBS solution at different pH, and the drug release experiment was performed at 37 °C and 150 rpm under constant temperature oscillation. Then, 0.5 mL samples were taken at 0.5, 1, 2, 4, 6, 8, 12, and 24 h, respectively, and supplemented with an equal volume of release medium. The obtained samples were centrifuged at 13,000 rpm for 10 min at 4 °C (13,000 rpm, 10 min), and the supernatant was diluted with an equal volume of hydrochloric acid (*v*/*v* = 20%). The concentration of ABZ was determined by UV-VIS spectrophotometer at 295 nm absorption wavelength.

The release of ABZ@MOFs under the action of different enzymes was investigated. A certain amount of pancreatic protease, pancreatic lipase, and α-Amylase were dissolved in normal saline to prepare the enzyme solution with equal concentration. A total of 3 mg ABZ@MOFs was dissolved in 35 mL of different enzyme solutions, and the drug release experiment was carried out at 37 °C under constant temperature oscillation at 150 rpm. Then, 0.5 mL samples were taken at 0.5, 1, 2, 4, 6, 8, 12, and 24 h, respectively, and supplemented with an equal volume of release medium. The obtained samples were centrifuged at 13,000 rpm for 10 min at 4 °C (13,000 rpm, 10 min), and the supernatant was diluted with an equal volume of hydrochloric acid (*v*/*v* = 20%). The concentration of ABZ was determined by UV-VIS spectrophotometer at 295 nm absorption wavelength.

(3) $$\mathrm{Cumulative}\;\mathrm{release}\;\mathrm{rate}\;\left(\%\right)=\frac{V\sum_{0}^{t-1}C_{t}+{\;V}_{0}C_{t}}{m_{\mathrm{drug}}}\times 100\%$$

where V is the volume of liquid medicine per sample; V_0_ is the total volume of the releasing medium; C_t_ is the drug concentration measured by UV at the corresponding time point. m_drug_ is the total drug mass loaded by ABZ@MOFs.

### 3.16. Transmembrane Transport Study

Caco-2 cells were cultured in DMEM high-glucose complete medium with 10% fetal bovine serum and 1% double antibody in an incubator containing 5% CO_2_ at 37 °C. Caco-2 cells with logarithmic growth phase were digested with 0.25% pancreatic protease, then prepared into single-cell suspension with complete medium and inoculated into 96-well culture plates with 5 × 10^4^ cells per well. After 24 h incubation in the incubator, 10 μL of different concentrations of drugs were added to each well. After 24 h incubation in the incubator, 10 μL of CCK-8 reagent was added to each well. The light absorption value of each well was measured at the wavelength of 450 nm by an enzyme-labeled instrument, and the cell survival rate was calculated:

(4) $$\mathrm{Survival}\;\mathrm{rate}\;\left(\%\right)=\frac{{\mathrm{OD}}_{P}{\;-\;\mathrm{OD}}_{B}}{{\mathrm{OD}}_{N}{\;-\;\mathrm{OD}}_{B}}$$

where OD_P_ is the OD value of the experimental group; OD_N_ is the OD value of the negative control group. OD_B_ is the OD value of the blank control group.

Caco-2 cells were inoculated into a 12-well Transwell culture plate with 2 × 10^5^ cells per well, in which 0.5 mL cell suspension was added to the top side (AP side) of the Transwell plate, and 1.5 mL complete medium was added to the base side (BL side). The single-layer model of Caco-2 cells was established after culture at 37 °C, 5% CO_2_ and saturated humidity for 21 days. According to the cytotoxicity results, the high, medium, and low concentrations of ABZ@MOFs were determined. To successfully establish the Caco-2 single-layer cell model, different concentrations of ABZ@MOFs were added to the AP side of the Transwell plate, and 1.5 mL of PBS was added to the BL side. After being cultured for 30, 60, 90, and 120 min in an incubator at 37 °C and 5% CO_2_, samples were taken from the BL side and centrifuged at 13,000 rpm for 5 min. The sample concentration was detected by high-performance liquid chromatography (HPLC). The *P_app_* and ER values of the transmembrane permeability of the drug were calculated according to the formula.

(5) $$P_{app}=\frac{\mathrm{dQ}/\mathrm{dt}}{A\;\cdot \;C_{0}}$$

(6) $$\mathrm{ER}=\frac{P_{app\;(\mathrm{BL}\to \mathrm{AP})}}{P_{app\;(\mathrm{AP}\to \mathrm{BL})}}$$

where dQ/dt is the penetration rate per unit time; A is the surface area of the polycarbonate film is 1.12 (cm^2^); C_0_ is the initial concentration of the drug (μg·mL^−1^); $P_{app\;(\mathrm{BL}\to \mathrm{AP})}$ is the transmembrane permeability from the BL side to the AP side; $P_{app\;(\mathrm{AP}\to \mathrm{BL})}$ is the transmembrane permeability from the AP side to the BL side.

Chromatographic conditions: A Diamonsil C18 (250 mm × 4.6 mm × 5 µm) column was used. Methanol/water (80:20) was used as the mobile phase with a flow rate of 1 mL·min^−1^. Detection wavelength 295 nm; column chamber temperature 30 °C; sample size 20 µL.

### 3.17. Bioavailability of ABZ@MOFs

Eighteen Wistar rats were selected and divided into three groups, with half of them being male and the other half being female. Each group was given a dose of 25 mg·kg^−1^ of ABZ by intragastric administration. Blood samples of 200–500 μL were collected from each rat at 0.5, 1, 2, 4, 6, 8, 10, 12, and 24 h after administration and were placed into pre-prepared anticoagulant centrifuge tubes. The samples were centrifuged at 13,000 rpm for 10 min, and the upper plasma was collected and stored at −80 °C in an ultra-low temperature refrigerator.

For sample preparation, plasma was thawed, and 50 μL of plasma was mixed with 150 μL of methanol. The mixture was vortexed for 1 min and then centrifuged at 13,000 rpm for 10 min. The supernatant (135 μL) was collected, followed by the addition of 200 ng·mL^−1^ mebendazole as the internal standard. The sample was vortexed for 1 min and then filtered through a microporous membrane. The drug content was quantified using UHPLC-MS/MS (UltiMate 3000 and Q-Exactive Orbitrap MS, Thermo Scientific, USA). Pharmacokinetic parameters were calculated using DAS 2.0 software.

Chromatographic conditions: An Accucore aQ UHPLC column (150 mm × 2.1 mm × 2.5 μm) was used. The column temperature was maintained at 40 ± 1 °C, and the sample tray was kept at 15 ± 0.5 °C. The mobile phase consisted of acetonitrile (A) and water with 0.1% formic acid (B). The elution gradient was as follows: 0–5 min, 95% B. The injection volume was 1 μL, and the flow rate was set to 0.3 mL·min^−1^.

Mass spectrometry conditions: Electrospray ionization (ESI) was used with high-purity nitrogen (purity > 99.9%) as the carrier gas. The sheath gas flow rate was 35 Arb, and the auxiliary gas flow rate was 10 Arb. The capillary temperature was set to 320 °C, with the ion lens (S-lens RF level) voltage frequency at 60. The spray voltage was 3.0 kV (positive mode), and full MS positive switching scanning mode was employed, with a scanning range of 150–800.

### 3.18. Statistical Analysis

Statistical analyses were conducted using SPSS 27.0 software. One-way ANOVA was used to compare differences between groups. Independent samples *t*-tests were performed to compare the two groups, and the non-parametric rank sum test was used for non-normally distributed data. Results are presented as the mean ± standard deviation (SD) from at least three independent replicates. Differences were considered statistically significant when *p* < 0.05.

## 4. Conclusions

In this study, three metal–organic frameworks (MOFs)—MOF-802, UiO-66-NH_2_, and MIL-125-NH_2_—were synthesized, and the poorly soluble drug ABZ was loaded into each framework. The properties of the MOFs and the corresponding ABZ@MOF formulations were characterized using various techniques. The results revealed distinct differences in particle size, morphology, and crystal structure among the three MOFs. In the three ABZ@MOFs, ABZ@MIL-125-NH_2_ exhibited the highest drug loading, followed by ABZ@UiO-66-NH_2_ and ABZ@MOF-802. The in vitro release behavior of the ABZ@MOFs was evaluated under different pH conditions and enzymes. All three formulations exhibited a dual pH- and enzyme-responsive release profile, indicating their ability to release ABZ under specific physiological conditions. Transmembrane transport studies further demonstrated that ABZ@UiO-66-NH_2_ outperformed ABZ@MIL-125-NH_2_ in terms of permeability. Finally, in vivo pharmacokinetic studies were conducted for ABZ@UiO-66-NH_2_ and ABZ@MIL-125-NH_2_. The results showed a significant increase in the area under the blood concentration-time curve (AUC_0–∞_) for both formulations compared to ABZ alone. Specifically, the AUC_0–∞_ of ABZ@UiO-66-NH_2_ and ABZ@MIL-125-NH_2_ increased by approximately 10.3-fold and 1.8-fold, respectively. In conclusion, MOFs effectively enhance the solubility and bioavailability of ABZ, making them promising candidates for use in oral drug delivery systems for ABZ.

## Figures and Tables

**Table 1:** Textural properties of MOFs and ABZ@MOFs.

	BET Surface Area (m^2^·g^−1^)	Pore Volume (mL·g^−1^)
MOF-802	238.71	0.18
ABZ@MOF-802	184.58	0.15
UiO-66-NH_2_	1179.78	0.55
ABZ@UiO-66-NH_2_	684.77	0.57
MIL-125-NH_2_	1103.10	0.74
ABZ@MIL-125-NH_2_	484.15	0.68

**Table 2:** Pharmacokinetic parameters of ABZ and ABZ@MOFs in rats (*n* = 6).

	ABZ	ABZ@UiO-66-NH_2_	ABZ@MIL-125-NH_2_
*C*_max_ (μg·L^−1^)	34.11 ± 5.19	81.21 ± 17.97 *	77.20 ± 15.26 *
*T*_max_ (h)	2.00 ± 0.38	1.83 ± 0.40	2.00 ± 0.14
t_1/2_ (h)	2.00 ± 0.38	29.70 ± 17.44 **	4.89 ± 1.95 **
CL (L·h^−1^·kg^−1^)	0.09 ± 0.01	16.61 ± 12.16 **	65.25 ± 18.20 ****
AUC_0–t_ (μg·L^−1^·h)	216.00 ± 27.55	702.6 ± 54.83 *	389.82 ± 111.67 *
AUC_0–∞_ (μg·L^−1^·h)	219.91 ± 25.27	2269.66 ± 707.32 *	409.82 ± 115.74 *
MRT_0–t_ (h)	6.16 ± 0.36	8.46 ± 2.78	5.90 ± 1.47
MRT_0–∞_ (h)	6.55 ± 0.37	37.89 ± 22.20 **	7.27 ± 1.95

Note: **** *p* < 0.0001, ** *p* < 0.01, * *p* < 0.05, compared with ABZ.

## Data Availability

The datasets used or analyzed during the current study are available from the corresponding author upon reasonable request.

## Supplementary Materials

The following supporting information can be downloaded at: https://www.mdpi.com/article/10.3390/ph18060819/s1, Supplementary Materials and Methods, and Figure S1: Synthesis of ABZ@MOFs; Figure S2: Cytotoxicity of ABZ@UiO-66-NH_2_ (A) and ABZ@MIL-125-NH_2_ (B) on Caco-2 cells; Table S1: ABZ@MOF-802 in vitro drug release model fitting equation; Table S2: ABZ@UiO-66-NH_2_ in vitro drug release model fitting equation; Table S3: ABZ@MIL-125-NH_2_ in vitro drug release model fitting equation.

## References

[1] Mcmanus D.P., Zhang W., Li J., Bartley P.B. (2003). Echinococcosis. Lancet.

[2] WHO WHO Estimates of the Global Burden of Foodborne Diseases: Foodborne Diseases Burden Epidemiology Reference Group 2007–2015 [EB/OL] (2015-12-01). https://www.who.int/publications/i/item/9789241565165.

[3] Nunnari G., Pinzone M.R., Gruttadauria S., Celesia B.M., Madeddu G., Malaguarnera G., Pavone P., Cappellani A., Cacopardo B. (2012). Hepatic echinococcosis: Clinical and therapeutic aspects. World J. Gastroenterol..

[4] Gil-Grande L.A., Rodriguez-Caabeiro F., Prieto J.G., Sánchez-Ruano J.J., Brasa C., Aguilar L., Garcίa-Hoz F., Casado N., Bárcena R., Alvarez A.I. (1993). Randomised controlled trial of efficacy of albendazole in intra-abdominal hydatid disease. Lancet.

[5] Wang S., Ma Y., Wang W., Dai Y., Sun H., Li J., Wang S., Li F. (2022). Status and prospect of novel treatment options toward alveolar and cystic echinococcosis. Acta Trop..

[6] Dong C.L., Zheng S.D., Liu Y.Y., Cui W.Q., Hao M.Q., God’spower B.O., Chen X.Y., Li Y.H. (2020). Albendazole solid dispersions prepared using PEG6000 and Poloxamer188: Formulation, characterization and in vivo evaluation. Pharm. Dev. Technol..

[7] Li C., Zhang Y., Pang M., Zhang Y., Hu C., Fan H. (2024). Metabolic mechanism and pharmacological study of albendazole in secondary hepatic alveolar echinococcosis (HAE) model rats. Antimicrob. Agents Chemother..

[8] Yan H., Zhong X., Liu Y. (2024). Improving the Solubility, Stability, and Bioavailability of Albendazole through Synthetic Salts. Molecules.

[9] Maqbool F., Moyle P.M., Tan M.S.A., Thurecht K.J., Falconer J.R. (2018). Preparation of albendazole-loaded liposomes by supercritical carbon dioxide processing. Artif. Cells Nanomed. Biotechnol..

[10] Li H., Song T., Shao Y., Aili T., Ahan A., Wen H. (2016). Comparative Evaluation of Liposomal Albendazole and Tablet-Albendazole Against Hepatic Cystic Echinococcosis: A Non-Randomized Clinical Trial. Medicine.

[11] Sawatdee S., Atipairin A., Sae Y.A., Srichana T., Changsan N., Suwandecha T. (2019). Formulation Development of Albendazole-Loaded Self-Microemulsifying Chewable Tablets to Enhance Dissolution and Bioavailability. Pharmaceutics.

[12] Abulaihaiti M., Wu X.W., Qiao L., Lv H.L., Zhang H.W., Aduwayi N., Wang Y.J., Wang X.C., Peng X.Y. (2015). Efficacy of Albendazole-Chitosan Microsphere-based Treatment for Alveolar Echinococcosis in Mice. PLoS Negl. Trop. Dis..

[13] Bhujbal S.V., Mitra B., Jain U., Gong Y., Agrawal A., Karki S., Taylor L.S., Kumar S., Zhou Q. (2021). Pharmaceutical amorphous solid dispersion: A review of manufacturing strategies. Acta Pharm. Sin. B..

[14] Patel K., Shah S., Patel J. (2022). Solid dispersion technology as a formulation strategy for the fabrication of modified release dosage forms: A comprehensive review. Daru.

[15] Peng T., Xu W., Li Q., Ding Y., Huang Y. (2022). Pharmaceutical liposomal delivery-specific considerations of innovation and challenges. Biomater. Sci..

[16] Chatterjee B., Almurisi S.H., Dukhan A.A.M., Mandal U.K., Sengupta P. (2016). Controversies with self-emulsifying drug delivery system from pharmacokinetic point of view. Drug Deliv..

[17] Wu M., Yang Y. (2017). Metal-Organic Framework(MOF)-Based Drug/Cargo Delivery and Cancer Therapy. Adv. Mater..

[18] Zhuang J., Kuo C., Chou L., Liu D., Weerapana E., Tsung C.K. (2014). Optimized metal-organic-framework nanospheres for drug delivery: Evaluation of small-molecule encapsulation. ACS Nano.

[19] Wang Z., Ma Y., Jiang Y., Zhou F., Wu Y., Jiang H., Wang R., Xu Q., Hua C. (2022). Encapsulating quercetin in cyclodextrin metal-organic frameworks improved its solubility and bioavailability. Sci. Food Agric..

[20] Han J., Xiao B., Le P.K., Mangwandi C. (2023). Enhancement of the Solubility of BS Class II Drugs with MOF and MOF/GO Composite Materials: Case Studies of Felodipine, Ketoprofen and Ibuprofen. Materials.

[21] Lv N., Guo T., Liu B., Wang C., Singh V., Xu X., Li X., Chen D., Gref R., Zhang J. (2017). Improvement in thermal stability of sucralose by γ-cyclodextrin metal-organic frameworks. Pharm. Res..

[22] Lin W., Hu Q., Jiang K., Yang Y., Yang Y., Cui Y., Qian G. (2016). A porphyrin-based metal-organic framework as a pH-responsive drug carrier. J. Solid State Chem..

[23] Jiang K., Ni W., Cao X., Zhang L., Lin S. (2022). A nanosized anionic MOF with rich thiadiazole groups for controlled oral drug delivery. Mater. Today Bio.

[24] Li L., Han S., Zhao S., Li X., Liu B., Liu Y. (2020). Chitosan modified metal-organic frameworks as a promising carrier for oral drug delivery. RSC Adv..

[25] Teplensky M.H., Fantham M., Li P., Wang T.C., Mehta J.P., Young L.J., Moghadam P.Z., Hupp J.T., Farha O.K., Kaminski C.F. (2017). Temperature treatment of highly porous zirconium-containing metal-organic frameworks extends drug delivery release. J. Am. Chem. Soc..

[26] Cai W., Gao H., Chu C., Wang X., Wang J., Zhang P., Lin G., Li W., Liu G., Chen X. (2017). Engineering phototheranostic nanoscale metal–organic frameworks for multimodal imaging-guided cancer therapy. ACS Appl. Mater. Interfaces.

[27] Zhang F., Dong H., Zhang X., Sun X., Liu M., Yang D., Liu X., Wei J. (2017). Postsynthetic modification of ZIF-90 for potential targeted codelivery of two anticancer drugs. ACS Appl. Mater. Interfaces.

[28] Leng X., Dong X., Wang W., Sai N., Yang C., You L., Huang H., Yin X., Ni J. (2018). Biocompatible Fe-based micropore metal-organic frameworks as sustained-release anti⁃cancer drug carriers. Molecules.

[29] Alsaedi M.K., Alothman G.K., Alnajrani M.N., Alsager O.A., Alshmimri S.A., Alharbi M.A., Alawad M.O., Alhadlaq S., Alharbi S. (2020). Antibiotic Adsorption by Metal-Organic Framework (UiO-66): A Comprehensive Kinetic, Thermodynamic, and Mechanistic Study. Antibiotics.

[30] Ahmadpour N., Sayadi M.H., Homaeigohar S. (2020). A hierarchical Ca/TiO/NH-MIL-125 nanocomposite photocatalyst for solar visible light induced photodegradation of organic dye pollutants in water. RSC Adv..

[31] Tripathi S., Sreenivasulu B., Suresh A., Rao C.V.S.B., Sivaraman N. (2020). Assorted functionality-appended UiO-66-NH2 for highly efficient uranium(VI) sorption at acidic/neutral/basic pH. RSC Adv..

[32] Dan-Hardi M., Serre C., Frot T., Rozes L., Maurin G., Sanchez C., Férey G. (2009). A new photoactive crystalline highly porous titanium(IV) dicarboxylate. J. Am. Chem. Soc..

[33] Yu Z., Fan W., Wang L., Qi J., Lu Y., Wu W. (2019). Effect of surface charges on oral absorption of intact solid lipid nanoparticles. Mol. Pharm..

[34] Sun J., Zhang L., Wang J., Feng Q., Liu D., Yin Q., Xu D., Wei Y., Ding B., Shi X. (2015). Tunable rigidity of (polymeric core)-(lipid shell) nanoparticles for regulated cellular uptake. Adv. Mater..

[35] Ijaz I., Gilani E., Nazir A., Bukhari A. (2020). Detail review on chemical, physical and green synthesis, classification, characterizations and applications of nanoparticles. Green Chem. Lett. Rev..

[36] Saka R., Chella N. (2021). Nanotechnology for delivery of natural therapeutic substances: A review. Environ. Chem. Lett..

