# Supplementary material for: Preparation and Evaluation of an Oral Administration System of Albendazole-Metal-Organic Framework Based on Dual Response to pH and Enzymes
Source: Pharmaceuticals (Basel). 2025 May 29;18(6):819. doi: 10.3390/ph18060819 (PMC12195660; doi:10.3390/ph18060819)
Supplement: Supplementary file 1 [file pharmaceuticals-18-00819-s001.zip › pharmaceuticals-3631681-supplementary.pdf]

Supplementary Materials

Preparation and Evaluation of an Oral Administration System of Albendazole-Metal-Organic Framework Based on Dual Response to pH and Enzymes

Weiqi Liu [1], Zhimei Guo [1], Yong Zhang [2], Yufei Guo [1], Ting Wang [3], Dahuan Liu [3] and Chunhui Hu [1,*]

1 College of Pharmacy, Qinghai University, Xining 810001, China

2 College of Clinical Medical, Qinghai University, Xining 810001, China

3 State Key Laboratory of Organic-Inorganic Composites, Beijing University of Chemical Technology, Beijing 100029, China

* Corresponding Author:
Chunhui Hu: chunhuihu@qhu.edu.cn

Supplementary Materials and Methods

S3.15.1 Drug Release Kinetics

The results of the cumulative drug release of the indicator components were fitted to the model by zero-order kinetic model, first-order kinetic model and Higuchi model respectively. This process yielded the relevant model equations and the correlation coefficients (R^2), and the goodness of fit was judged by R^2.

S3.16.1 Cytotoxicity of Caco-2 Cells

Caco-2 cells were cultured in DMEM high-glucose complete medium with 10% fetal bovine serum and 1% penicillin/ streptomycin in an incubator containing 5% CO_2 at 37 °C. The logarithmic-phase Caco-2 cells were digested with 0.25% trypsin, followed by pipetting into a single cell suspension using a complete culture medium and seeded in a 96-well culture plate (5×10^4 cells/well, 100 μL/well). Following 24 h of cultivation in the incubator, 10 μL of drugs of different concentrations were added to each well, and cells were cultured for an additional 24 h. Then, 10 μL of CCK-8 reagent was added to each well, and then measuring their OD_{450} using a microplate reader. The cell survival rates of cells treated with different drugs at different concentrations were calculated according to Formula (S1).

$$\text{Survial rate (\%)} = \frac{OD_P - OD_B}{OD_N - OD_B} \quad \text{(S1)}$$

OD_P is the OD value of the experimental group; OD_N was the OD value of negative control group. OD_B is the OD value of the blank control group.

Table S1. ABZ@MOF-802 *in vitro* drug release model fitting equation

Release Condition	Model	Simultaneous Equations	R^2
pH = 1.2	zero-order release	$M_t = 0.0060t + 0.0712$	0.7371
	first-order release	$M_t = 0.1802\ (1 - e^{-0.2426t})$	0.9221
	Higuchi model	$M_t = 0.0371t^{1/2} + 0.0274$	0.9148
pH = 6.8	zero-order release	$M_t = 0.0102t + 0.1425$	0.5605
	first-order release	$M_t = 0.3197\ (1 - e^{-0.3116t})$	0.9740
	Higuchi model	$M_t = 0.0660t^{1/2} + 0.0609$	0.7966
pH = 7.4	zero-order release	$M_t = 0.0449t + 0.0120$	0.8636
	first-order release	$M_t = 0.3979\ (1 - e^{-0.0649t})$	0.8686
	Higuchi model	$M_t = 0.067t^{1/2} - 0.0261$	0.8509
Protease	zero-order release	$M_t = 0.0046t + 0.1092$	0.6788
	first-order release	$M_t = 0.1724\ (1 - e^{-0.7728t})$	0.7884
	Higuchi model	$M_t = 0.0291t^{1/2} + 0.0743$	0.8867
Lipase	zero-order release	$M_t = 0.0056t + 0.0997$	0.7440
	first-order release	$M_t = 0.1829\ (1 - e^{-0.4672t})$	0.7909
	Higuchi model	$M_t = 0.0345t^{1/2} + 0.0590$	0.9270
Amylase	zero-order release	$M_t = 0.0064t + 0.0309$	0.8832
	first-order release	$M_t = 0.1864\ (1 - e^{-0.2409t})$	0.7955
	Higuchi model	$M_t = 0.1794t^{1/2} - 0.1062$	0.9866

Note: M_t is the cumulative release degree at time t. t is the time of drug release.

Table S2. ABZ@UiO-66-NH_2 *in vitro* drug release model fitting equation

Release Condition	Model	Simultaneous Equations	R^2
pH = 1.2	zero-order release	$M_t = 0.0006t + 0.0208$	0.4840
	first-order release	$M_t = 0.0422（1 - e^{-0.2481t}）$	0.9438
	Higuchi model	$M_t = 0.0060t^{1/2} + 0.0116$	0.7387
pH = 6.8	zero-order release	$M_t = 0.0138t + 0.1402$	0.6976
	first-order release	$M_t = 0.3952（1 - e^{-0.2236t}）$	0.9760
	Higuchi model	$M_t = 0.0863t^{1/2} + 0.0370$	0.8984
pH = 7.4	zero-order release	$M_t = 0.0202t + 0.2148$	0.6162
	first-order release	$M_t = 0.5731（1 - e^{-0.2565t}）$	0.9736
	Higuchi model	$M_t = 0.1288t^{1/2} + 0.0575$	0.8428
Protease	zero-order release	$M_t = 0.0089t + 0.1407$	0.7488
	first-order release	$M_t = 0.2803（1 - e^{-0.3847t}）$	0.8459
	Higuchi model	$M_t = 0.0548t^{1/2} + 0.0761$	0.9295
Lipase	zero-order release	$M_t = 0.120t + 0.1255$	0.8133
	first-order release	$M_t = 0.3510（1 - e^{-0.204t}）$	0.8474
	Higuchi model	$M_t = 0.0720t^{1/2} + 0.0431$	0.9432
Amylase	zero-order release	$M_t = 0.0171t + 0.3212$	0.6076
	first-order release	$M_t = 0.5945（1 - e^{-0.2709t}）$	0.6981
	Higuchi model	$M_t = 0.1095t^{1/2} + 0.1874$	0.8341

Note: M_t is the cumulative release degree at time t. t is the time of drug release.

Table S3. ABZ@MIL-125-NH_2 *in vitro* drug release model fitting equation

Release Condition	Model	Simultaneous Equations	R^2
pH = 1.2	zero-order release	$M_t = 0.0041t + 0.0891$	0.5757
	first-order release	$M_t = 0.1519（1 - e^{-0.5237t}）$	0.8719
	Higuchi model	$M_t = 0.0264t^{1/2} + 0.0565$	0.8142
pH = 6.8	zero-order release	$M_t = 0.0030t + 0.0895$	0.52533
	first-order release	$M_t = 0.1316（1 - e^{-0.8103t}）$	0.8208
	Higuchi model	$M_t = 0.0194t^{1/2} + 0.0652$	0.7769
pH = 7.4	zero-order release	$M_t = 0.0036t + 0.0358$	0.6848
	first-order release	$M_t = 0.1005（1 - e^{-0.2350t}）$	0.9744
	Higuchi model	$M_t = 0.0225t^{1/2} + 0.0089$	0.8885
Protease	zero-order release	$M_t = 0.0057t + 0.0474$	0.8275
	first-order release	$M_t = 0.1627（1 - e^{-0.1607t}）$	0.9326
	Higuchi model	$M_t = 0.0339t^{1/2} + 0.0087$	0.9531
Lipase	zero-order release	$M_t = 0.0129t + 0.1215$	0.8024
	first-order release	$M_t = 0.3669（1 - e^{-0.1951t}）$	0.9487
	Higuchi model	$M_t = 0.0782t^{1/2} + 0.0308$	0.9563
Amylase	zero-order release	$M_t = 0.0063t + 0.0754$	0.8469
	first-order release	$M_t = 0.1829（1 - e^{-0.4672t}）$	0.7909
	Higuchi model	$M_t = 0.0345t^{1/2} + 0.0590$	0.9270

Note: M_t is the cumulative release degree at time t. t is the time of drug release.

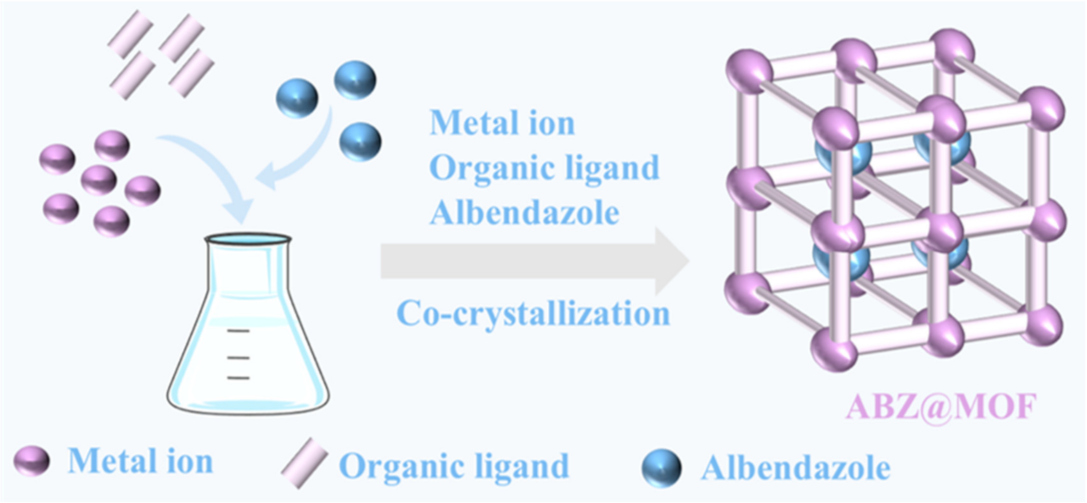

Figure S1. Synthesis of ABZ@MOFs.

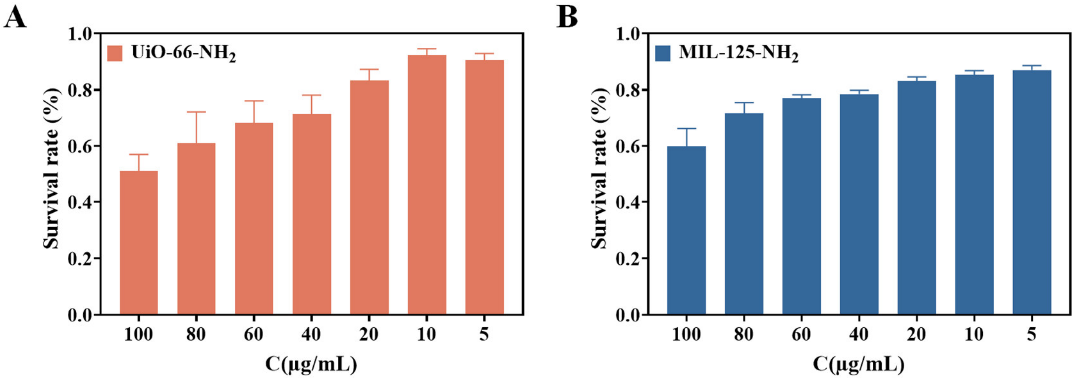

Figure S2. Cytotoxicity of ABZ@UiO-66-NH_2 (**A**) and ABZ@MIL-125-NH_2 (**B**) on Caco-2 cells.
